# Obesity Influences on Patients With Non-valvular Cardiomyopathy in Relation to Early In-Hospital Outcomes and Health System Burden

**DOI:** 10.7759/cureus.24859

**Published:** 2022-05-09

**Authors:** Ghada Shalaby, Kawlah Samarin, Renan Alabbasi, Amani A Fallatah, Tala Roblah, Rahaf A Abdulwahab, Rawan N Althomali, Emad M Babateen, Faisal Y Alhodian, Sheeren Khaled

**Affiliations:** 1 Faculty of Medicine, Zagazig University, Zagazig, EGY; 2 Cardiac Center, King Abdullah Medical City, Makkah, SAU; 3 College of Medicine, Umm Al-Qura University, Makkah, SAU; 4 College of Medicine, Ibn Sina National College for Medical Studies, Jeddah, SAU; 5 College of Medicine, King Abdulaziz University, Jeddah, SAU; 6 College of Medicine, King Khalid University, Abha, SAU; 7 College of Medicine, King Saud Bin Abdulaziz University for Health Sciences, Jeddah, SAU; 8 Cardiology, Banha University, Banha, EGY

**Keywords:** body mass index: bmi, non-valvular cardiomyopathy, in-hospital outcome, health system, obesity

## Abstract

Background

Our study aimed to assess the burden of obesity on the health system and outcomes in patients with non-valvular cardiomyopathy.

Methods

A retrospective analytical cohort, single-center study was conducted at King Abdullah Medical City (KAMC), Makkah, from June 2019 to June 2020, and includes all non-valvular cardiomyopathy (NVCM) patients. The patients were divided into two groups, obese (BMI*≥*30) and non-obese (BMI<30). The two groups were compared using a t-test and a chi-squared test for continuous and categorical data and regression analysis.

Results

A single-center, retrospective study was conducted at KAMC, Makkah, and included all NVCM patients (ejection fraction or EF*≤*45%) who were admitted during this study period. A total of 626 NVCM patients were included in this cohort study; they had a mean BMI of 29*±*8.1 and a mean EF of 28.4*±*9.7. Patients were divided into two groups, obese (BMI*≥*30) and non-obese (BMI<30). Obese patients represented 37% (n=231) of our study population. The non-ischemic category of cardiomyopathy had a higher prevalence among the obese (35% vs 27%). A higher percentage of obese patients presented with heart failure (HF) symptoms rather than ischemia or arrhythmia (46%, 40%, and 7% for HF symptoms, ischemia, and arrhythmia, respectively). There was no significant difference in NVCM complications, including cardiogenic shock, pulmonary edema, and cardiac arrest, between the two groups. Obese patients had a significantly higher post-myocardial infarction (MI) ejection fraction (29.7±9.7 vs 27.5±9.7, p=0.01). We found a statistically significant positive correlation between BMI and length of in-hospital stay (P=0.04). In-hospital mortality was non-significantly different between our two groups, although numerically, it was higher among the non-obese group (obesity paradox) (10% vs 12%, p=0.2). Type of cardiomyopathy, cerebrovascular stroke, smoking, and sacubitril/valsartan intake were detected as independent predictors of in-hospital mortality among our patients.

Conclusions

Obesity among NVCM patients sets more burden on health facilities by the prolongation of the in-hospital stay of patients although BMI is not an independent predictor of death in those patients.

## Introduction

In the last several decades, cardiomyopathies have become a more prevalent cause of heart failure in young individuals [1]. When heart failure gets worse, the length of time patients stay in the hospital, the cost of care, and the healthcare system's burden all go up [2]. Several clinical and laboratory indicators may be used to determine the prognosis of heart failure patients, one of which is obesity, which is defined as a body mass index (BMI) of more than or equal to 30 [3].

Obesity is a risk factor for heart failure and an important prognostic factor [4]. Obesity has become more common in recent decades, with 68% of US people being classified as overweight or obese [1]. Despite the fact that obesity is associated with increased all-cause mortality, after adjusting for established cardiovascular risk factors, such as body mass index (BMI) and waist circumference (WC), obesity is only marginally associated with coronary heart disease and stroke [4-6]. There is conflicting data on whether greater BMI is associated with an increased risk of heart failure or not [7-8]. Obesity, especially severe obesity, can cause hemodynamic changes that dispose of cardiac morphology and ventricular function changes [9]. These changes comprise cardiac left ventricular hypertrophy, output increase, diastolic and systolic dysfunction, including both ventricles, and may predispose to left and right heart failure, which may be exacerbated by obesity-related comorbidities, such as hypertension, sleep apnea/obesity hypoventilation syndrome, and neurohormonal and metabolic modifications, a condition known as obesity cardiomyopathy [10].

The majority of studies have documented an inverse relationship between obesity and heart failure; this concept is that overweight and mild-to-moderately obese patients have a better prognosis and lower risk of complications than normal-weight patients, a concept termed the obesity paradox [11]. Weight loss seems to be the only way to get rid of obesity-related comorbidities [12]. Healthcare investigators and workers should pay attention to the prevention of obesity and the treatment of affected individuals [13].

The obesity pandemic has left non-negligible bills to be paid by those affected, health services, and communities [13]. The correlation between obesity and NVCM was not well-assessed. In Saudi Arabia, limited studies have assessed the association between body weight/body mass index (BMI) and the in-hospital outcomes among patients with Non-Valvular Cardiomyopathy. so, we aimed in this study to estimate the burden of obesity on the health system and in-hospital outcomes of patients with non-valvular cardiomyopathy.

This article was previously presented as a poster at the American College of Cardiology’s 70th Annual Scientific Session & Expo (ACC.21) in Atlanta, GA, on May 17, 2021.

## Materials and methods

Study design

This is a retrospective analytical cohort study that included 626 non-valvular cardiomyopathies (NVCM) patients admitted at King Abdullah Medical City (KAMC) during the period from June 2019 to June 2020.

Participants

We included all patients admitted to the cardiac center at KAMC with an established diagnosis of NVCM from June 2019 to June 2020. Those patients who fit the inclusion eligibility criteria in KAMC records were included. we exclude any patients with insufficient data and any patients outside the scope of the service of KAMC.

Ethical approval

Our study was designed to be part of the standards of patient care to investigate and improve the quality of NVCM prognoses and outcomes among a diverse population. Consent was taken, and the study received approval from the ethics committee/institutional review board (IRB) of our institution, King Abdullah Medical City, Mecca, Saudi Arabia (No. H-02- K-001).

Data collection

All demographic data were collected (including age, gender, nationality, height, weight, and residency status (Pilgrim, Resident, or Saudi). Risk factors, including diabetes mellitus (DM), hypertension (HTN), smoking, dyslipidemia, presence of chronic kidney disease (CKD), old cerebrovascular accidents (CVA), chronic obstructive lung disease (COPD), peripheral vascular disease (PVD), and atrial fibrillation (AF), were identified from the history and case note. Clinical presentation (heart failure symptoms, ischemia, or arrhythmias), Laboratory results (including hemoglobin (HB), blood urea nitrogen (BUN), creatinine, serum sodium (Na), serum potassium (K), troponin, glycosylated hemoglobin (HBA1c), brain natriuretic peptide (BNP), and vitamin D level) were obtained from the electronic and medical records. Echocardiography data (ventricular function, left ventricular (LV) thickness, LV, right ventricle (RV), and left atrium (LA) sizes, valvular assessment, left ventricular thrombus, and pericardial effusion) and coronary angiography findings (vessel involvement and procedure done) were also recorded. History of device insertion, including implantable cardioverter-defibrillator and cardiac resynchronization therapy (ICD/CRTD) and in-hospital outcome measures (including in-hospital death, cardiogenic shock, cardiac arrest, arrhythmias, pulmonary edema, ventilation, and length of hospital stay) were reviewed and recorded. Management strategies used for our patients, including medical treatment and revascularization therapy (percutaneous coronary intervention (PCI) or coronary artery bypass grafting (CABG)) were also identified. Discharge medications, including sacubitril/valsartan (Entresto), angiotensin-converting enzyme inhibitors (ACEi), antiplatelet (aspirin and clopidogrel), beta-blockers, statins, diuretics (including loop diuretics, spironolactone, and metolazone), digoxin, nitrates, and digoxin were also recorded.

Definitions and group division

Concerning the etiology of NVCM, the diagnosis was as follows:

I - Dilated cardiomyopathy (DCM): Left ventricular ejection fraction (LVEF) <0.45 (>2SD) as well as a left ventricular end-diastolic diameter >117% of the estimated value adjusted for age and body surface according to the Henry equation: (45·3(BSA) 1/3"0·03(age)"7·2), which corresponds to two SD of the predicted normal limit +5%).

II - Ischemic cardiomyopathy (ICM): Diagnostic criteria were similar to DCM in addition to coronary artery disease obstruction (≥50% narrowing of the diameter of the lumen of the left main coronary artery or ≥70% narrowing of the diameter of the lumen of the left anterior descending coronary artery, left circumflex artery, or right coronary artery).

III - Hypertrophic cardiomyopathy (HCM): In the absence of secondary causes of hypertrophy (HTN, aortic stenosis), it is diagnosed based on ≥15 mm wall thickness in one or more myocardial segments measured by echocardiography. The BMI is calculated by dividing a person's weight in kilograms by the square of their height in meters. The World Health Organization (WHO) states that for adults, obesity is defined as a body mass index of 30 or greater. Our study’s patients were divided into two groups according to BMI: group 1 obese (BMI≥30) and group 2 non-obese (BMI<30), and we compared both groups concerning all mentioned data.

Statistical analysis

Statistical analysis was performed using the Statistical Package for the Social Sciences (SPSS) software package (SPSS Inc.; Chicago, IL), version 21.0. Data are presented as mean ± SD for continuous data and percentage for categorical variables. Univariate analysis was done using the t-test or chi-squared test for continuous and categorical data, respectively. P-value ≤0.05 was considered significant and not significant if it is >0.05. Correlations and regression analysis were also calculated.

## Results

A total of 626 consecutive NVCM patients were admitted to our cardiac center during the period from 2019 to 2020. They were divided into two groups: non-obese NVCM (395 patients (63%)) and obese NVCM (231 patients (37%)). We categorized our data into main five categories.

Baseline characteristics and clinical data

As shown in Table 1, the Saudis were more obese compared to non-Saudi patients (P=0.08). Age, cardiovascular risk factors, residency status, and associated morbidities were found to be statistically non-significant in both the non-obese and obese groups of patients. The obese patients had less prevalence of ischemic cardiomyopathy (ICM) as an etiology of heart failure (63% vs 71%% for obese and non-obese, respectively) but a higher prevalence of DCM (35% vs 27% for obese and non-obese, respectively). Concerning the prevention of sudden cardiac death and improving both quality of life as well as mortality, the utilization of device therapy (ICDs/CRTDs) was significantly higher among obese NVCM patients (P=0.029).

**Table 1:** Baseline demographic and clinical data of non-obese and obese NVCM patients NVCM: non-valvular cardiomyopathy; DM: diabetes mellitus; COPD: chronic obstructive pulmonary disease; PVD: peripheral vascular disease; ICD: implantable cardiac defibrillator; CRTD: cardiac resynchronization therapy defibrillator

Variable	Non-obese 395 (63%)	Obese 231 (37%)	P-value
Age, Years (Mean ± SD)	56.76±13.49	55.21± 12.47	0.345
Male gender, N (%)	327 (83%)	178 (77%)	0.091
Saudi nationality, N (%)	284 (72%)	192 (83%)	0.081
Pilgrims, N (%)	45 (11.4%)	14 (6%)	0.132
DM, N (%)	249 (63%)	145 (62.7%)	0.242
HTN, N (%)	257 (65%)	157 (68%)	0.760
Dyslipidemia, N (%)	103 (26%)	51 (22%)	0.636
Smoking, N (%)	134 (34%)	90 (39%)	0.512
Chronic kidney disease, N (%)	55 (14%)	39 (17%)	0.396
Cerebrovascular accident, N (%)	67 (17%)	51 (22%)	0.266
COPD, N (%)	20 (5%)	16 (7%)	0.705
PVD, N (%)	16 (4%)	17 (7%)	0.400
Atrial fibrillation, N (%)	60 (15%)	25 (11%)	0.455
Ischemic cardiomyopathy, N (%)	281 (71%)	145 (63%)	0.140
Dilated cardiomyopathy, N (%)	106 (27%)	81 (35%)	0.140
Hypertrophic cardiomyopathy, N (%)	8 (2%)	5 (2%)	0.140
Device insertion (ICD/CRTD)	43 (11%)	40 (17%)	0.029

Clinical presentation, laboratory findings, and in-hospital outcome measures

As shown in Table 2, the obese patients showed higher presentation with heart failure symptoms rather than ischemia and arrhythmias (46%, 40%, and 7% vs 37%, 54%, and 5%; P=0.014 for obese and non-obese, respectively). There was potentially lower in-hospital mortality among obese patients (10% vs 12%; P=0.25). Other in-hospital outcome measures, including cardiogenic shock, cardiac arrest, and pulmonary edema, were also slightly lower among obese compared to non-obese patients.

**Table 2:** Clinical presentation and in-hospital outcomes of non-obese and obese NVCM patients NVCM: non-valvular cardiomyopathy

Variable	Non-obese 395 (63%)	Obese 231 (37%)	P value
Congestive heart failure, N (%)	146 (37%)	106 (46%)	0.014
Ischemic symptoms (chest pain), N (%)	213 (54%)	92 (40%)	
Arrhythmias on presentation, N (%)	20 (5%)	16 (7%)	
Others, N (%)	16 (4%)	17 (7%)	
In hospital death, N (%)	48 (12%)	23 (10%)	0.254
Cardiogenic shock N (%)	23 (6%)	7 (3%)	0.133
Cardiac arrest, N (%)	24 (6%)	9 (4%)	0.585
Arrhythmias during hospitalization, N (%)	83 (21%)	51 (22%)	0.457
Pulmonary edema, N (%)	31 (8%)	14 (6%)	0.694
Intubated/Ventilated, N (%)	24 (6%)	13 (5.6%)	0.612
Length of hospital stay (Mean± SD)	13.12±39.757	15.61±48.204	0.015

Table 3 represents the values of laboratory markers that were identified among obese NVCM patients (P=0.09, 0.06, 0.01, 0.07, and 0.07 for hemoglobin on discharge, serum creatinine, peak troponin, glycated hemoglobin (HBA1C), and brain natriuretic peptide (BNP), respectively).

**Table 3:** Laboratory findings of non-obese and obese NVCM patients NVCM: non-valvular cardiomyopathy; BUN: blood urea nitrogen; HbA1C: glycated hemoglobin; BNP: brain natriuretic peptide

Test name	Non-obese 395 (63%)	Obese 231 (37%)	P-value
HB on admission (mg/dl), Mean ± SD	13.32 ± 2.08	13.99 ± 10.73	0.125
HB on discharge (mg/dl), Mean ± SD	12.79 ± 2.09	12.77 ± 2.32	0.096
Creatinine (mg/dl), Mean ± SD	2.09 ± 1.077	1.50 ± 1.45	0.069
BUN (mg/dl) ), Mean ± SD	21.14 ± 12.645	20.73 ±14.457	0.393
Serum sodium (mg/dl), Mean ± SD	135.97 ± 10.718	138.11 ± 8.57	0.654
Serum potassium (mg/dl), Mean ± SD	4.22 ± .567	4.26 ± 0.632	0.185
Troponin (mg/dl), Mean ± SD	47.96 ± 271.962	15.19 ± 60.308	0.017
HbA1C (mg/dl), Mean ± SD	7.78 ± 2.386	7.69 ± 2.010	0.076
BNP (pg/ml), Mean ± SD	1159.09 ± 2529.3	919.76 ± 1202.5	0.07
Vitamin D (ng/ml), Mean ± SD	20.31 ± 13.311	15.54 ± 15.030	0.729

**Figure 1:**
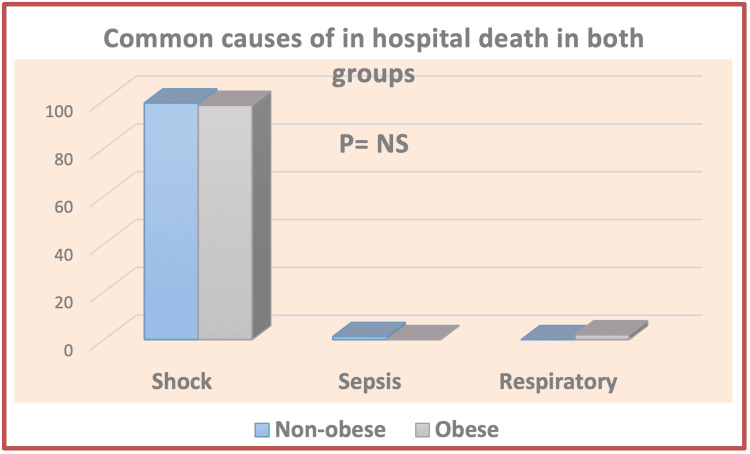
Common causes of in-hospital death in both groups

It can be seen from the data in Figure 1 that cardiogenic shock is the most common recorded cause of mortality among our patients.

Cardiac imaging data

Obese NVCM patients showed a significantly higher mean LV ejection fraction such as 29.72±9.78 vs 27.52±9.73; P=0.012 but worse RV dysfunction (P=0.018) compared to the non-obese group of patients. Other echocardiography parameters, including LV and LA sizes, valvular dysfunction, LV thrombus, and pericardial effusion, were all found to be statistically non-significant in both groups (Table 4).

**Table 4:** Echocardiography of non-obese and obese NVCM patients NVCM: non-valvular cardiomyopathy; LVEF: left ventricular ejection fraction; RV: right ventricle; LV: left ventricle; LA: left atrium; MR: mitral regurgitation

Variable	Non-obese 395 (63%)	Obese 231 (37%)	P-value
LVEF (Mean ± SD)	27.52±9.73	29.72±9.78	0.012
Diastolic dysfunction grade II/III, N (%)	138 (35%)	90 (39%)	0.390
Thickened LV	28 (7%)	21 (9%)	0.488
LV dilation, N (%); Moderate; Severe	48 (12%) 75 (19%)	32 (14%) 39 (17%)	0.902
Moderate/Severe RV dilation, N (%)	32 (8%)	30 (13%)	0.186
Moderate/Severe RV dysfunction, N (%)	44 (11%)	42 (18%)	0.018
Moderate/Severe LA dilation, N (%)	94 (24%)	49 (21%)	0.684
MR grade III/IV, N (%)	103 (26%)	69 (30%)	0.272
TR grade III/IV, N (%)	79 (20%)	32 (14%)	0.222
Left ventricular thrombus, N (%)	36 (9%)	23 (10%)	0.902
Pericardial effusion, N (%)	20 (5%)	9 (4%)	0.430

Nevertheless, obese patients had a significantly lower incidence of both single and multi-vessel disease (P<0.05) (Table 5).

**Table 5:** Angiographic data and management strategies of ischemic cardiomyopathy subgroup of non-obese and obese NVCM patients NVCM: non-valvular cardiomyopathy; LM: left main; LAD: left anterior descending; LCX: left circumflex; RCA: right coronary artery; MVD: coronary microvascular disease; PCI: percutaneous coronary intervention; PCTA: percutaneous transluminal coronary angioplasty; CABG: coronary artery bypass graft

Variable	Non-obese ICM 281 (45%)	Obese ICM 145 (23%)	P-value
LM disease, N (%)	19 (6.8%)	9 (6.2%)	0.840
LAD disease, N (%)	169 (60%)	68 (47%)	0.008
LCX disease, N (%)	109 (39%)	32 (22%)	0.000
RCA disease, N (%)	110 (39%)	39 (27%)	0.006
MVD, N (%)	135 (48%)	48 (33%)	0.001
Medical treatment, N (%)	56 (20%)	74 (51%)	0.008
PCI/PTCA, N (%)	138 (49%)	41 (28%)	
CABG, N (%)	87 (31%)	31 (21%)	

Management

Obese patients showed significantly higher medical therapy utilization and lower revascularization management compared to non-obese NVCM patients (P=0.008) (Table 5).

Regarding medical treatment, obese patients were more candidates for sacubitril/valsartan (Entresto) (P=0.062) and potentially were in higher need of different types of diuretics as compared to the non-obese group of patients (Table 6).

**Table 6:** Discharge medications of non-obese and obese NVCM patients NVCM: non-valvular cardiomyopathy; ACEi: angiotensin-converting enzyme inhibitor; ARB: angiotensin receptor blocker

Variable	Non-obese 395 (63%)	Obese 231 (37%)	P-value
Sacubtril/valsartan (Entresto), N (%)	103 (26%)	79 (34%)	0.062
ACEi/ARBs, N (%)	241 (61%)	120 (52%)	0.052
Aspirin, N (%)	336 (85%)	192 (83%)	0.460
Clopidogrel, N (%)	245 (62%)	125 (54%)	0.070
Beta-blockers, N (%)	363 (92%)	206 (89%)	0.352
Statin, N (%)	312 (79%)	188 (81%)	0.662
Loop diuretics, N (%)	304 (77%)	180 (78%)	0.726
Spironolactone, N (%)	268 (68%)	162 (70%)	0.613
Metolazone, N (%)	20 (5%)	14 (6%)	0.570
Nitrates, N (%)	107 (27%)	56 (24%)	0.494
Digoxin, N (%)	12 (3%)	14 (6%)	0.086

Regression and correlations

Ischemic cardiomyopathy as an etiology of NVCM, history of cerebrovascular stroke, smoking, and intake of sacubitril/valsartan were the independent predictors of mortality among our patients. BMI was not found to be an independent predictor of in-hospital death in the current study (Table 7).

**Table 7:** Binary regression analysis of independent predictors of in-hospital mortality ICM: ischemic cardiomyopathy

Variable	B	S.E	Exp (B)	P-value
Age	-0.269	0.323	0.764	0.405
BMI	0.002	0.025	1.002	0.951
Smoking	0.046	0.019	1.047	0.013
Type of cardiomyopathy (ICM)	0.033	0.011	1.034	0.003
Cerebrovascular stroke	0.899	0.455	0.407	0.048
Sacubitril/Valsartan	0.530	0.313	1.698	0.050

Most importantly, there was a significant positive correlation between BMI and in-hospital length of stay (p=0.04).

## Discussion

King Abdullah Medical City is the only cardiac center in the region providing tertiary care services, such as revascularization and advanced heart failure therapy, and hence receives most of the NVCM patients for proper evaluation, workup, and further advanced management. Moreover, its location in the holy city of Makkah is unique, as it receives a considerable number of Hajj patients and residents of different nationalities. This allowed us to study that diverse population; it also reflects a significant burden for the provided service and hospital costs. All these factors contributed to the creation of the current study. To our knowledge, no study has been done in Saudi Arabia to see how BMI affects NVCM patients and the healthcare system when they are being treated in a hospital.

We included around 626 patients diagnosed with NVCM who were referred and admitted to our cardiac center from June 2019 to June 2020, with 37% obese patients and 63% non-obese.

Saudi patients with heart failure were more obese compared to non-Saudis. The different genetic, racial, environmental, and cultural backgrounds of non-Saudis who came from various places might explain this finding. There was no statistical difference between the two groups in terms of their age or gender, as well as their risk factors like diabetes, hypertension, and smoking.

Obese patients with NVCM exhibited lower in-hospital mortality rates than non-obese NVCM patients (obesity paradox). This was prescribed in previous studies conducted to assess the relationship between BMI and mortality in HF patients [14-18]. Our findings cope with the obesity paradox that was discussed before in cardiac patients, generally including ischemic heart and HF patients. However, it demonstrates an inverse relationship between BMI and in-hospital mortality rates; we do not know if this relationship can be applied to morbidly obese patients (BMI>35 kg/m2). Multiple mechanisms can explain the obesity paradox in HF patients. The catabolic state that occurs in HF patients with obesity represents the metabolic reserve, and high levels of serum lipoproteins associated with increased body fat in obese patients may also play an anti-inflammatory role, neutralizing circulating bacterial endotoxins or cytokines [19]. Some theories have been used to explain the obesity paradox as the early presentation of obese patients with less complex disease, and multiple comorbidities cause early recognition and treatment of the patients [17,20]. The higher value of hemoglobin on discharge, lower values of serum creatinine, HBA1C, BNP, and peak troponin, and significantly lower incidence of multi-vessel disease in NVCM obese patients might help explain potentially favorable outcomes among them.

A nationwide cohort study to evaluate the short-term effect of obesity in hospitalized patients with HF was published in 2020, included more than 1.5 million HF patients, and concluded that obesity was not associated with a higher incidence of inpatient mortality; however, it prolonged in-hospital stay in hospitalized patients with HF [21]. Our study’s finding was per this, as there was a significant positive correlation between BMI and in-hospital length of stay. This might be explained by the complexity of their disease and the higher need for HF management.

Although LVEF tended to be higher in obese patients, similar results to Harrison et al. [22], RV systolic function was more affected among them. This can be due to obesity-related pulmonary diseases, which may cause more burden on right ventricular performance, including obstructive sleep apnea, episodes of subclinical hypoxemia, high risk of recurrent DVT, and pulmonary embolization. Moreover, our findings suggest that the obese have a paradoxically lower coronary artery disease burden and severity. This again implies the presence of the "obesity paradox" and might be explained by the possible referral of obese patients to coronary angiography earlier than their non-obese counterparts.

In the current study, BMI was not recognized as an independent predictor for in-hospital death. This agreed with multiple studies that acknowledged that obese patients had significantly lower in-hospital and overall mortalities [22-23]. Obesity was not an independent predictor of overall mortality after multivariate analysis. In-hospital mortality in the current study was independently predicted by the type of cardiomyopathy (ischemic cardiomyopathy), smoking, and history of CVA, and this highlights different risk predictors that might affect prognosis in patients with different characteristics. The use of sacubitril/valsartan was also identified as a predictor of mortality among our patients, and this reflects the appropriate utilization of this drug in those patients with advanced disease, taking into consideration that its long-term efficacy is not tested in the current study due to a lack of follow up.

Interestingly, regarding the etiology and management of NVCM, obese patients had less prevalence of ICM and higher prevalence of DCM; this explains why obese patients presented to the hospital with congestive heart failure manifestations more often than other manifestations, with a higher need for device therapy in the form of ICD or CRTD and requiring more sacubitril/valsartan use. These results were synonymous with one large, longitudinal, population-based study whose main findings were that increased BMI was linked with an increased risk of heart failure in both genders, and this risk increased with BMI rise [7,24].

There was no significant difference in other outcomes of HF, including pulmonary edema, cardiac arrest, arrhythmias, dyspnea, and cardiogenic shock, between the two BMI categories. Our data are consistent with other published data [16]. Finally, the results of our study should give the heart failure research community a focused view of obesity’s impact on in-hospital outcomes in different types of NVCM and how an advanced HF treatment, including sacubitril/valsartan and device therapy, is effectively approached and used in candidate patients. Our research also highlights the burden of obesity on the health system, including a tertiary center with a considerable volume of variable population and a high flow rate.

Limitations

Part of the study was conducted during the coronavirus disease 2019 (COVID-19) pandemic. In addition, the number of enrolled patients is limited due to the nature of the single-center study and the selection of NVCM patients only, so the results can’t be generalizable. Moreover, there was no follow-up data or long-term outcomes since some patients were pilgrims or non-Saudis, and others lost their follow-up in the hospital. As our study includes patients with EF less than or equal to 45, we had patients with heart failure with preserved ejection fraction (HFpEF). The influence of obesity on both groups should be studied separately in the coming studies. We hope to reduce the effect of this limitation by motivating more hospitals to conduct similar research to create multicenter, larger sample studies in the future.

## Conclusions

Although BMI was not an independent predictor of in-hospital mortality in patients with NVCM, obesity prolongs the in-hospital stay of NVCM patients, which causes less bed availability and more burden on the health care system. Most of the obese patients presented with heart failure symptoms rather than ischemic symptoms or arrhythmia and had a lower prevalence of MVD. In-hospital mortality was lower in obese patients but not statistically significant. The type of cardiomyopathy, cerebrovascular stroke, smoking, and sacubitril/valsartan intake were the independent predictors of in-hospital mortality. There is a need for prospective studies to elucidate the mechanisms of this relationship.

Identification and awareness of the mortality and morbidity predictors in NVCM patients might lessen the burden and save time and money for society. This study highlights the necessity of obesity management since it’s a heavy burden on both the health system and financial resources. Another reason is that it was found that obesity prolongs in-hospital stay. This study also suggests that other research addressing obesity with a larger sample size should be conducted to further explore the mortality and morbidity predictors in patients with NVCM. It is worth mentioning that health care systems should pay attention not only to obesity prevention but also to the treatment of patients with full medical and financial support, which in turn will prevent a significant burden on health system resources.
